# Supplementary material for: Causal interplay between lactose intolerance and gut microbiota: a combined bidirectional Mendelian randomization and in vivo validation study
Source: Front Nutr. 2026 Jun 1;13:1803337. doi: 10.3389/fnut.2026.1803337 (PMC13265576; doi:10.3389/fnut.2026.1803337)
Supplement: Supplementary file 1 [file Data_Sheet_1.zip › supplementary materials/Forward/forest plot/ebi-a-GCST90027706.finngen_R12_E4_LACTONAS.pdf]

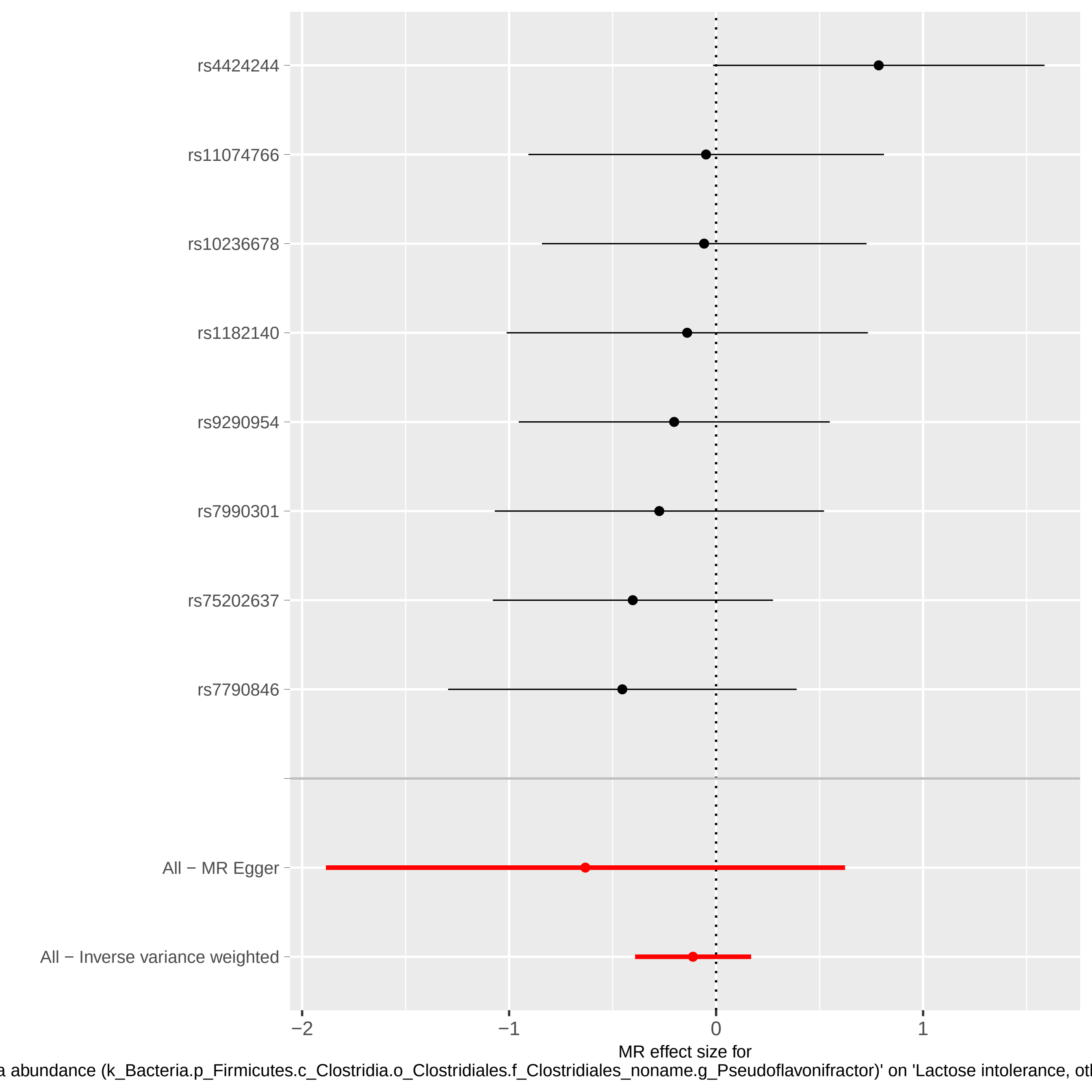
rs4424244
rs11074766
rs10236678
rs1182140
rs9290954
rs7990301
rs75202637
rs7790846
All − MR Egger
All − Inverse variance weighted
−2
−1
0
1
MR effect size for
a abundance (k_Bacteria.p_Firmicutes.c_Clostridia.o_Clostridiales.f_Clostridiales_noname.g_Pseudoflavonifractor)' on 'Lactose intolerance, otl
